# Severe Anaemia during Late Pregnancy

**DOI:** 10.1155/2012/485452

**Published:** 2012-09-04

**Authors:** Mahenaz Akhtar, Ismail Hassan

**Affiliations:** ^1^School of Medicine, Keele University, Staffordshire ST5 5BG, UK; ^2^Consultant Obstetrics & Gynaecology, University Hospital of North Staffordshire NHS Trust, Maternity Building, City General Hospital, Newcastle Road, Stoke-on-Trent ST4 6QG, UK

## Abstract

Vitamin B12 deficiency is uncommon in pregnancy, it occurs in 10–28% of uncomplicated pregnancies, and is associated with a few complications. We present a case report of a 21-year-old patient with severe anaemia during late pregnancy caused by vitamin B12 deficiency. At 38 weeks gestation and with a BMI of 48.9, a history of rupture of membranes was given but not confirmed. On examination, she appeared pale and therefore full blood counts were done. Interestingly her haemoglobin (Hb) levels were 3.7 g/dL. Folate and vitamin B12 levels were also found to be low, and the diagnosis of anaemia caused by vitamin B12 deficiency was made. After treatment with vitamin B12 injections, folic acid and blood transfusions, the patient's haemoglobin levels improved from 3.7 g/dL to 10.7 g/dL. The conclusion is that effective history taking, diagnosis, and management can prevent many complications that are usually associated with vitamin B12 deficiency anaemia.

## 1. Introduction

Anaemia during pregnancy is common and has both maternal and foetal consequences [1]. The most common cause is iron deficiency anaemia, other causes include infection, folate, and vitamin B12 deficiency [1, 2]. A diagnosis of anaemia can be made if haemoglobin levels are less than 11.0 g/dL in the last trimester of pregnancy [1]. Anaemia specifically caused by vitamin B12 deficiency occurs in 10–28% of uncomplicated pregnancies [3]. Vitamin B12 can only be obtained from animal products [4]. More than 1000 *μ*g of vitamin B12 is stored in fertile women eating a mixed diet [4]. At term, foetal vitamin B12 stores should be 25–50 *μ*g [5]. 20% of women show a physiological drop in vitamin B12 levels during pregnancy, with lowest levels reached at third trimester [3, 4, 6]. We report the case of a 21-year-old with severe anaemia caused by vitamin B12 deficiency at 38 weeks gestation. 

## 2. Case Report

A 21-year-old Caucasian woman, with an BMI of 48.9, gravida 2 para 1, presented at 38 weeks and 2-days gestation with a 2 day history of vaginal leak suggestive of rupture of membranes but this was not confirmed by speculum or ultrasound scan. The presenting complaint also included feeling unwell, vomiting, and abdominal pain over 3 weeks. Past obstetric history included an induced vaginal delivery 2 years previously.

On observation, BP was 130/67 mmHg, heart rate was 101 beats per minute (bpm), temperature was 37.3°C, oxygen saturation was 97%, and respiratory rate was 16 breaths per minute, and she was also noted to look rather pale. 

Blood samples were taken and showed an Hb of 3.7 g/dL (normal 11.5–16.5 g/dL) and platelet count of 126 × 10^9^/L (normal 150–400 × 10^9^/L). A blood film showed right shifted neutrophils, occasional reactive lymphocytes and tear drop poikilocytes. At this point anaemia caused by B12 and folate deficiency was highly suspected and more bloods were tested. These blood tests showed an exceptionally high LDH at 4449 u/L, high ferritin levels at 654 ng/mL, serum B12 levels were low at 180 pg/mL (200–900 pg/mL), and serum folate levels were low at 0.6 ng/mL (3–12 ng/mL). Blood tests also showed decreased levels of potassium, creatinine, calcium, albumin, and urea. There were increased levels of alkaline phosphatise, aspartate transaminase (AST), and bilirubin. A differential diagnosis of vitamin B12 and folate deficiency anaemia was to be supported.

**Figure 1:**
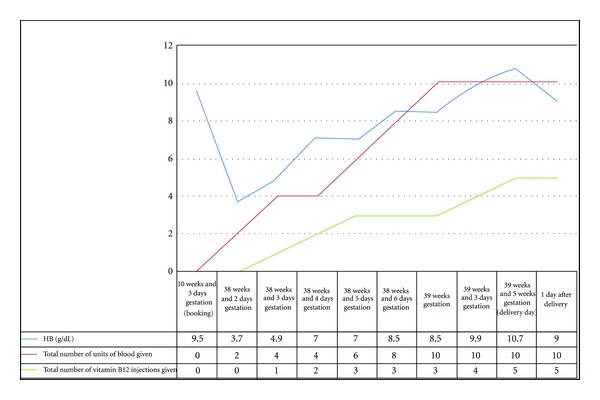
Graph showing patients' haemoglobin levels, and total number of units of blood and vitamin B12 injections given.

She was admitted onto the ward, and over the course of the next 11 days was given treatment to resolve the vitamin B12 deficiency anaemia.  Figure 1 summarises the treatment carried out and investigations.

As well as blood transfusions and vitamin B12 injections, 200 mg ferrous sulphate, folic acid, and multivitamin tablets were given orally until the delivery of the baby. 

As growth was below the 10th centile and there was reduced liquor, it was decided that labour would be induced at 39 weeks and 5 days gestation. The stages of labour from onset of contractions to delivery of the placenta occurred lasted 78 minutes. A live female baby was delivered by spontaneous vaginal delivery, and there were no complications to the mother after delivery.

The female baby weighed 2820 grams, which was below the tenth centile. Apart from this, the baby was otherwise well, with no other complications.

The following day blood results showed maternal Hb level of 9.0 g/dL and platelet level of 332 × 10^9^/L. The baby was feeding well (bottle fed), had passed stool and urine; there were no concerns. On neonatal examination, the findings were normal except for a “clicky hip” for which a referral was made. The patient was discharged home with no problems at time of discharge. At follow-up home visits, there were no concerns for the patient or baby.

## 3. Discussion

Vitamin B12 deficiency is most likely to occur in the third trimester, particularly in women with inadequate diets [2]. Vitamin B12 is involved in the folate pathway in the formation of DNA, which is essential for cell multiplication during pregnancy that is required for foetal development [3, 4]. Hyperhomocysteinemia, which is caused by low vitamin B12 levels, is associated with many clinical conditions, for example, placental vasculopathy, which has an effect on foetal growth [4, 7]. 

Maternal vitamin B12 determines foetal vitamin B12 levels [7]. During pregnancy, vitamin B12 is transferred from mother to foetus by active transport across the placenta into foetal circulation which results in foetal serum level being double that of maternal serum levels [5, 8]. 

The typical symptoms of megaloblastic anaemia were found to be vomiting, diarrhoea, and pyrexia, with oedema and albuminuria occurring most often in the later stages [9]. In our case, the only typical symptom was vomiting. In severe cases, and as in our case, white blood cell and platelet levels, drop, the patients' presenting platelet levels were 126 × 10^9^/L [10].

Anaemia is diagnosed by firstly determining haematocrit or haemoglobin (Hb) levels; in the last trimester the cut-off point is 11.0 g/dL (1,11). The next step is to determine the exact cause of the anaemia with additional tests [1, 11]. With vitamin B12 deficiency anaemia, additional tests involve measuring urinary methylmalonic acid and serum vitamin B12 levels [5, 10]. However, there is no gold standard for diagnosing this anaemia [12].

The treatment of vitamin B12 deficiency anaemia involves loading doses of daily to weekly parenteral injections, consisting of hydroxocobalamin 1 mg, over 1 to 3 months to replenish body stores of vitamin B12, this is then followed by a maintenance regimen [10, 12]. 

Anaemia has many maternal complications including cardiovascular symptoms, reduced physical and mental performances, reduced immune function and fatigue [1]. Foetal consequences include growth retardation, prematurity, intrauterine death, amnion rupture, neural tube defects, and low birth weight [1, 2]. The only complication in our case was low birth weight which was below the 10th centile at 2820 g.

Reduced maternal vitamin B12 deficiency is commonly caused by reduced dietary intake, especially with vegetarian diets [5]. Our patient's diet consisted of only chips and cheese throughout pregnancy. The patient had no appreciation of a balanced diet, including knowledge of different food groups. Nutritional status during pregnancy, especially in late pregnancy, has been linked to the birthweight of the baby [7]. Vitamin B12 deficiency has been associated with low birthweight as was in our case [7]. 

Vitamin B12 deficiency can cause anencephaly [2, 13]. Schorah et al. found very low vitamin B12 levels in 3 anencephalic mothers compared with controls; this may be due to the fact that vitamin B12 is involved in the metabolism of neural tissue [13]. Pathological changes that occur due to vitamin B12 deficiency are demyelination, axonal degeneration, and neuronal death [5]. Elevation in homocysteine and reduced tetrahydrofolate, as well as secondary elevation of guanidinoacetate (neurotoxic) may be involved in the pathogenesis of encephalopathy [5].

Vitamin B12 deficiency is also an independent risk factor for foetal neural tube defects [2]. The underlying mechanism involves the homocysteine pathway by elevation in plasma total homocysteine [8]. 

This deficiency has also been associated with recurrent foetal loss and neonatal deaths [2, 9]. Bennet found that severe deficiency caused a high incidence of recurrent foetal loss [2]. The cause of this recurrent foetal loss is two-fold: firstly due to damage of decidual and chorial vessels leading to abnormal placentation and secondly because of direct embryo toxicity from hyperhomocysteinemia [14]. Relationship between third trimester maternal anaemia and foetal death was particularly strong [11]. Other complications of vitamin B12 anaemia include preterm delivery and periods of infertility [2]. 

## 4. Conclusion

Anaemia caused by vitamin B12 deficiency is uncommon. This paper has shown that efficient and early diagnosis and management of vitamin B12 deficiency in the third trimester of pregnancy can prevent the complications that are usually associated with this type of anaemia.
